# Muscle function and fatigability of trunk flexors in males and females

**DOI:** 10.1186/s13293-017-0133-y

**Published:** 2017-04-17

**Authors:** Rita E. Deering, Jonathon W. Senefeld, Tatyana Pashibin, Donald A. Neumann, Sandra K. Hunter

**Affiliations:** Department of Physical Therapy, Marquette University, PO Box 1881, Milwaukee, WI 53201 USA

**Keywords:** Trunk flexors, Sex differences, Fatigability, Strength, Abdominal muscles

## Abstract

**Background:**

Optimal function of the abdominal muscles is necessary for several life functions including lifting and carrying tasks. Sex differences in strength and fatigability are established for many limb muscles and back extensor muscles, but it is unknown if sex differences exist for the abdominal muscles despite their functional importance.

**Methods:**

Eighteen females (24.3 ± 4.8 years) and 15 males (24.1 ± 6.6 years) performed (1) isometric trunk flexion maximal voluntary contractions (MVCs) in a range of trunk positions to establish a torque-angle curve and (2) submaximal (50% MVC), intermittent isometric contraction (6 s on, 4 s off) until task failure to determine fatigability of the trunk flexor muscles. Dual X-ray absorptiometry quantified body fat and lean mass. Physical activity levels were quantified with a questionnaire. Torque-angle curves, electromyography (EMG), MVC torque, and torque steadiness were compared with repeated measures ANOVA with sex as a between-subjects factor.

**Results:**

For the torque-angle curve, MVC torque was reduced as the trunk angle increased toward flexion (*p* < 0.001). Males had greater MVC torque than females at the extended positions (31% difference), with no sex differences in torque in upright sitting (*p >* 0.05). Time-to-task failure for the submaximal fatigability task in upright sitting was similar between males and females (12.4 ± 7 vs 10.5 ± 6 min). Time-to-task failure was positively associated with strength (*r* = 0.473, *p* = 0.005) and self-reported physical activity (*r* = 0.456, *p* = 0.030). Lean mass in the trunk was positively associated with trunk flexor strength (*r* = 0.378, *p* = 0.011) and self-reported physical activity (*r* = 0.486, *p* = 0.007). Finally, torque steadiness [coefficient of variation of torque (CV)] during submaximal isometric contractions decreased with contraction intensity and was similar for males and females across all intensities.

**Conclusions:**

Unlike many limb muscle groups, males and females had similar fatigability and torque steadiness of the trunk flexor muscles during isometric contractions. Stronger individuals, however, exhibited less fatigability. Lower self-reported physical activity was associated with greater fatigability of trunk flexor muscles. The relationship between strength and fatigability of the trunk flexor muscles and physical activity supports the importance of abdominal muscle strengthening to offset fatigability in both males and females.

## Background

Optimal function of the abdominal muscles is important for functional mobility, including lifting and carrying tasks [1]. While the abdominal muscles are the prime movers of trunk flexion [2], this muscle group performs multiple other key functions. For example, the abdominal muscles, along with the diaphragm and pelvic floor muscles, regulate intra-abdominal pressure (IAP) [3]. Through this regulation of IAP, the abdominal muscles also provide postural support and stability of the lumbar spine, while allowing transfer of loads from the extremities to the trunk (and vice versa) [1, 3–5]. The abdominal muscles also play a role in breathing and continence [1, 6–8], through synergistic action with the diaphragm and pelvic floor muscles. The abdominal muscles are also isometrically active during movements of the upper and lower extremities [4, 5, 9]. Due to the need for abdominal muscle activation during nearly all functional tasks, these muscles are often active isometrically and at submaximal levels during sustained contractions such as during a carrying task, or repetitive contractions, as during lifting tasks.

Given the importance of optimal abdominal muscle function and the lack of knowledge on the function of this muscle group, a more thorough understanding of the strength, fatigability, and force control of these muscles is required. Sex differences in strength and fatigability have been identified in the upper and lower limb muscles, with females typically demonstrating lower strength but decreased fatigability compared with males [10, 11]. Smidt et al. [12] showed decreased fatigability of the trunk flexor and extensor muscles in females during a maximal, reciprocal, dynamic fatiguing protocol; however, this study did not assess the contribution of the hip flexor muscles to the trunk flexion task. Similarly, females were less fatigable in the back extensor muscles compared with males for a sustained submaximal isometric contraction at 50% maximal voluntary isometric contraction (MVC) [13]. Intermittent isometric contractions of the abdominal muscles may be more functionally relevant, because postural stabilization is often achieved with isometric contractions, and many activities of daily living are repetitive in nature. The use of intermittent contractions also removes the confounding factor of the reduced blood flow experienced during sustained contractions. It is not known if there are sex differences in strength or fatigability for the trunk flexor muscles for a submaximal intermittent, isometric task.

An important aspect of force control that can affect functional performance is the steadiness of a contraction, which can be measured as the magnitude of force (or torque) fluctuations [14, 15]. During isometric contractions of limb muscles, the fluctuations in force are quantified as the standard deviation (SD) about a target force of a sustained contraction or as the coefficient of variation (CV) of torque when normalized to mean force produced [15]. In limb muscles, force fluctuations are primarily explained by oscillations in common drive to the motor neuron pool and the motor unit discharge rate variability [15–17] resulting in larger force fluctuations at higher contraction intensities when calculated as the SD of force. The force fluctuations expressed as the CV, however, are reduced at the higher intensities of contraction, creating an inverse relationship between force fluctuations and contraction force [18–20]. Whether this relationship between steadiness and contraction intensity is also present during trunk flexion contractions is unknown, particularly given that activation of the motor neuron pool occurs from multiple spinal levels during trunk flexion [2]. In addition to the input of common drive onto the motor neuron pool, other factors may influence the shape of this relationship for the abdominals. Additionally, in limb muscles, force fluctuations will increase throughout a fatiguing contraction [21, 22], and it is also unknown if this is true during fatiguing exercise of the trunk flexor muscles in males and females. Generally, at low intensities, females exhibit greater force fluctuations than males. This has been observed in the elbow flexors, first dorsal interosseous (finger abduction) and knee extensor muscles [23–25], but it is not known if sex differences in force steadiness exist for the trunk flexor muscles. A better understanding of the force control of the abdominal muscles would be beneficial because the abdominal muscles play a major role in regulation of intra-abdominal pressure and the stiffness of the spine [26], so it is possible that large fluctuations in abdominal muscle force could cause fluctuations in IAP, thus impacting spinal stiffness.

This study determined if there were sex differences in isometric trunk flexion for MVC torque across a range of trunk flexion angles, fatigability, and torque steadiness in young, healthy males and females. We *hypothesized* that males would generate greater peak isometric trunk flexion torque because males typically have a greater muscle mass than females, especially in the upper body [27]. Due to the critical role of the abdominal muscles in postural support and as accessory muscles of ventilation [2], we also hypothesized that, although females would be less fatigable than males, the sex differences would be small compared to those observed in limb muscles (e.g., elbow flexors) [11, 21, 28].

## Methods

Eighteen females (24.3 ± 4.8 years) and 15 males (24.1 ± 6.6 years) participated in two experimental sessions, separated by at least 1 day and no more than 1 week, to examine abdominal muscle function. All participants were healthy and free from cardiovascular disease, neurological impairment, chronic pain syndromes, and orthopedic conditions of the spine and lower extremities and did not use any medications that impact neurotransmitters and/or neuromuscular excitability. The female participants reported they had never been pregnant. All participants provided written informed consent. The protocol was approved by the Institutional Review Board at Marquette University, in accordance with the Declaration of Helsinki.

A dual X-ray absorptiometry (DXA) scan was performed during the first session to obtain estimates of lean body mass, fat mass, and bone mineral density of the whole body and specific regions using a GE Lunar iDXA (GE Healthcare, Little Chalfont, UK). Real-time ultrasound (GE Vivid e; 8 LRS linear probe) was used to assess thickness of the rectus abdominis muscles. The participants also completed a questionnaire to estimate physical activity [29]. The physical activity questionnaire involved recall of occupational and leisure physical activity over the previous 12 months, and each activity was weighted to estimate the metabolic cost of the activity (METs). The participants were provided with a list of 37 activities (with space for additional activities) and asked to provide frequency, quantity, and intensity of activities over the previous 12-month period. METs were also able to be estimated for occupational physical activity based on occupational history and questions. Thus, the weekly metabolic equivalents (MET h week^−1^) was calculated from the occupational and leisure physical activity. Laboratory measurements of isometric trunk flexion MVC torque, submaximal torque steadiness, and fatigability were made using a Biodex System 4 dynamometer (Biodex, Shirley, New York), as described below.

### Trunk flexion torque

**Fig. 1:**
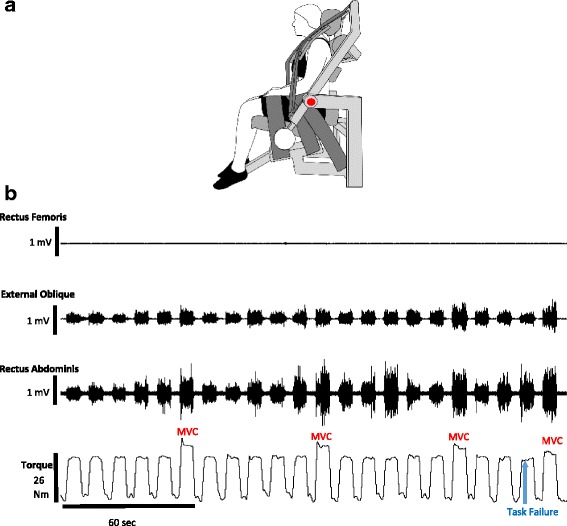
Experimental setup and representative data of fatigue task. **a** Experimental setup of the biodex back flexion/extension attachment in the upright sitting (0°) position, used for the fatigue task. The frame of the attachment is represented in *light gray*, with padding in *medium gray* and restraints in *dark gray*. The *red dot* indicates the axis of rotation of the device. **b** Representative trace of raw data for a young male. EMG traces for the right rectus femoris, left external oblique, and right rectus abdominis are shown. The bottom trace is trunk flexion torque and shows the 50% submaximal contractions with MVCs performed every minute starting at minute 6 for a 9.7 min fatiguing exercise bout

The participants were seated in a back flexion-extension attachment for a Biodex dynamometer (Fig. 1a) such that the right anterior superior iliac spine was aligned with the axis of rotation of the dynamometer. A scapular roll (15-cm diameter) was positioned at the level of the scapular spine, and the head rest of the device was adjusted to participant comfort. The pelvis was stabilized with a sacral pad posteriorly and two tightly fastened straps anteriorly. A strap was also used to restrain the thighs. Vertical straps were placed on the anterior aspect of each shoulder to restrain the upper body, and these straps were joined at the midline of the chest with a buckle. The participants were instructed to flex their trunk, as though performing an abdominal curl up, without allowing their legs to lift off of the seat. All trunk flexion attempts were visually assessed by the investigator (a physical therapist), and feedback was provided to the participants if compensatory movement patterns were observed.

Trunk flexion torque was recorded online using a Power 1401 A-D converter and Spike2 software [Cambridge Electronics Design (CED), Cambridge, UK]. Torque signals were digitized at 500 Hz and displayed on a 48-cm monitor placed ~150 cm in front of the participant.

### Electromyography

Electromyography (EMG) signals were obtained for the right rectus abdominis, left external oblique, and right rectus femoris using two 8-mm silver chloride surface recording electrodes (Coulbourn Instruments, Whitehall, PA) arranged in a bipolar configuration according to recommended placements [30]. EMG signals were amplified (1000×) and band-pass (13–1000 Hz) and Notch (60 Hz) filtered with Coulbourn modules (Coulbourn Instruments, Allentown, PA). Signals were recorded online using a Power 1401 A-D converter (CED) and were digitized at 2000 Hz.

### Experimental protocol

All participants were instructed to refrain from caffeine for at least 2 h and alcohol, pain medication, and anti-inflammatory medications for at least 12 h prior to experimental sessions.

### Session one

#### Body composition

A DXA scan was performed to estimate fat mass, lean muscle mass, and bone mineral density. The participants were asked to remove all metal before the scan. The participants were positioned on the scanner bed in a supine position with forearms in neutral position. Legs were bound just superior to the knees and the ankles with straps to prevent external rotation of the hips during the scan. The participants were asked to lie as still as possible and to not talk, unless there was a problem, during the scan.

#### Muscle thickness

Ultrasonography was used to determine thickness of the right rectus abdominis muscle. The participants were positioned in a supine position on a plinth with their shirt removed. Muscle thickness measurements of the right rectus abdominis [31] were taken at 2.5 cm above and below the umbilicus. The full medial-lateral width of the rectus abdominis was scanned at each of these positions and the measurement was taken, while the participant held their breath at end expiration, in the region that visually appeared to be the thickest.

#### Torque-angle curve

In order to assess MVC torque at varying muscle lengths and establish a torque-angle curve for the trunk flexor muscles, the participants were placed in six different positions within the back flexion/extension attachment (Biodex). A calibrated digital angle gauge (Wixey WR300 Digital Angle Gauge, Barry Wixey Development, Sanibel, FL) was used inferior to the sternal notch to ensure that each participant was at the same position. Upright sitting was identified as zero degrees. MVC isometric torque was evaluated at 20° of flexion, upright sitting, and 10°, 20°, 30°, and 40° of extension, in a randomized order.

#### Maximal voluntary contractions

The participants performed at least three isometric trunk flexion MVCs for ~3 s at each position. MVCs were separated by at least 1 min of rest, in order to limit fatigability. MVCs were performed, with verbal encouragement, until the participant was able to perform two contractions where torques were within 5% of each other. The higher of these two contractions was used as the MVC. For each MVC, the participant was asked to flex the trunk forward, as though curling the shoulders down toward the hips without engaging the lower extremities. The participants were closely examined while performing trunk flexion MVCs in order to identify evidence of movements involving other muscle groups (e.g., legs elevating slightly off of chair due to activation of hip flexor muscles), and trials were only included in analysis if correct form was performed by the participant. On average, four MVCs were performed at each trunk angle, with a range of 3 to 6 MVCs for most participants. One participant did require seven trials at one position but was able to successfully perform the correct trunk flexion maneuver in 3–5 attempts for the remaining trunk angles. The participants were also cued to not hold their breath.

Although flexion of the upper trunk is primarily performed by the rectus abdominis muscles [2], EMG of the rectus femoris muscle in the lower limb was measured to provide quantifiable evidence that lower extremity muscles, particularly those that contribute to hip flexion, were not being excessively utilized during the trunk flexion contractions. In order to normalize EMG of the rectus femoris during trunk flexion contractions, knee extension MVCs were performed to obtain maximal EMG. Three trials were performed at each position, with at least 1 min of rest in between each contraction. To perform MVCs of the knee extensors, an adjustable strap was placed around the shank of each participant to stabilize the limb.

#### Steadiness (torque fluctuations)

Submaximal isometric contractions at five different intensities (5, 10, 20, 50, and 70% MVC) were performed among a subgroup of participants (9 females, 11 males) in order to assess torque fluctuations (steadiness). The participants were positioned upright (0°) and a computer monitor provided visual feedback of a target line at the respective trunk flexion torque. The participants were instructed to trace the line as steady as possible for 6 s. Two trials were performed at each intensity, with intensities performed in a random order.

### Session two

#### Intermittent submaximal fatiguing protocol

The participants performed an intermittent isometric fatiguing protocol with the trunk flexor muscles in the upright position (0°), as many postural tasks are performed in upright positions, at an intensity of 50% MVC torque. Prior to the fatiguing exercise, the participants performed baseline MVCs for trunk flexion and knee extension, as previously described. For the fatiguing exercise of the trunk flexor muscles, a target line representing 50% MVC was displayed on a computer screen in front of the participant. Vertical cursors were displayed to cue the participants when to contract (6 s) and when to relax (4 s). The participants were instructed to trace the target line as accurately as possible during each separate contraction. A trunk flexion MVC was performed every minute and sustained for 6 s, in order to match the contraction/relaxation cycle of the fatiguing task. Every 60 s, the participants verbally rated their perceived exertion during the 50% MVC fatiguing task (modified Borg scale, 0–10 scale) [32].

Each participant was verbally encouraged to continue the fatiguing task as long as possible. Task failure was defined as inability to maintain target torque (50% MVC) for 3 of the 6 s of a contraction or an MVC ≤50% of baseline MVC. If task failure was reached during a submaximal contraction, an MVC was performed as the next contraction and then the fatiguing task was terminated. Representative torque and EMG activity of a fatiguing exercise bout is shown in Fig. 1b. To measure recovery, MVCs of the trunk flexor muscles were performed 10 and 20 min after the end of the intermittent submaximal fatiguing protocol.

### Data analysis

Data obtained from the Biodex (MVC torque, steadiness of contraction, submaximal torque) was analyzed offline using Spike 2 software (CED). The MVC torque during trunk flexion contractions was determined by averaging the force over a 0.5-s interval around the peak torque during the MVC. Time-to-task failure for the intermittent submaximal fatigue task was calculated from the onset of the first submaximal contraction to the end of the final MVC.

Torque steadiness was quantified as the standard deviation (SD) of torque during submaximal contractions and during the fatiguing protocol. Because the amplitude of the torque fluctuations is dependent on the absolute torque [15], steadiness was also quantified as the coefficient of variation (CV) of torque, calculated as (SD of torque/mean torque) × 100%. During the sets of submaximal isometric contractions (5, 10, 20, 50, and 70% MVC), torque steadiness was quantified over a 3-s interval of a 6-s contraction. In order to represent changes in the control of force during the intermittent submaximal fatigue task, torque steadiness was calculated as the average torque fluctuations from three submaximal contractions at each quartile of the exercise protocol (beginning, 25%, 50%, 75%, and 100% of time-to-task failure).

The maximal EMG activity of each muscle during trunk flexion MVCs was quantified as the root mean square (RMS) value during the same 0.5-s interval as the MVC torque. During the intermittent submaximal fatigue task, EMG was quantified as the average RMS of the EMG signal from three submaximal contractions and was obtained at the same intervals of the same submaximal contractions as torque and steadiness were calculated. MVCs for knee extension were also performed during each test session to obtain maximal EMG from the rectus femoris. Rectus abdominis and external oblique EMG during submaximal contractions was normalized to the RMS of the maximum EMG signal of each respective muscle obtained during trunk flexion MVCs in each test session. A 30-Hz high-pass filter was applied to rectus abdominis and external oblique EMG to remove ECG artifact [33]. EMG of rectus femoris during trunk flexion contractions (MVCs and submaximal contractions) was normalized to the RMS of the maximum EMG signal obtained from knee extension MVCs at each respective trunk position.

Ultrasound images were analyzed using the GE Vivid e ultrasound machine. Thickness of the right rectus abdominis muscle was measured from the inferior aspect of the superior fascial border to the superior aspect of the inferior fascial border [31].

### Statistical analysis

Data within the text and tables are presented as means ± SD and in figures as means ± standard error of the mean (SEM). Independent samples *t* tests were used to compare sex differences (males and females) for the following variables: subject characteristics, self-reported physical activity levels, rectus abdominis muscle thickness, MVC torque of trunk flexor muscles prior to fatiguing exercise, and time-to-task failure of the fatiguing task. Repeated measures analysis of variance (ANOVA) was used to compare across conditions with sex as a between subject factor for the following variables: torque steadiness (SD of torque and CV of torque) across time during the fatiguing task and across intensities (% MVC) for submaximal contractions, MVC torque at each trunk position (torque-angle curve), and MVC torque from task failure through recovery. Pearson correlation was performed to determine the associations between dependent variables, with only significant correlations reported. Statistical analysis was performed on SPSS version 24 (IBM, Armonk, NY, USA). Significant differences were defined as *p* ≤ 0.05.

## Results

### Baseline measures

Age, body mass index (BMI), and physical activity levels were similar for males and females (Table 1). Males were taller (*t*_31_ = −3.7, *p* = 0.001), weighed more (*t*_31_ = −2.0, *p* = 0.049), and had lower body fat (*t*_31_ = 7.4, *p* < 0.001) than females. Males also had greater lean mass in the trunk (*t*_31_ = −6.4, *p* < 0.001) than females, even when trunk lean mass was normalized to height (*t*_31_ = −6.1, *p* < 0.001). Thickness of the right rectus abdominis muscle belly was 1.4 times greater in males than females when measured at 2.5 cm above the umbilicus (*t*_31_ = −3.48, *p* = 0.003) and 1.3 times thicker in males than females at 2.5 cm below the umbilicus (*t*_31_ = −3.7, *p* = 0.002). See Table 1.

**Table 1:** Subject characteristics

		Females (*n* = 18)	Males (*n* = 15)
Age	Years	24.3 ± 4.8	24.1 ± 6.6
Weight	kg	65.2 ± 12.6	73.1 ± 8.8*
Height	cm	166.6 ± 8.4	176.8 ± 7.6*
Body mass index	kg m^−2^	23.3 ± 3.5	23.1 ± 2.3
Body fat	%	32.5 ± 5.1	18.6 ± 5.7*
Lean mass in trunk	kg	20.0 ± 3.6	27.0 ± 3.2*
Trunk lean mass/height	kg/cm	0.12 ± 0.02	0.15 ± 0.02*
Self-reported physical activity over the preceding 12 months (data from 16 females and 14 males)	Met h week^−1^	44.7 ± 27.4	60.1 ± 39.9
Rectus abdominis muscle thickness (above umbilicus)	cm	1.0 ± 0.18	1.4 ± 0.36*
Rectus abdominis muscle thickness (below umbilicus)	cm	0.97 ± 0.13	1.3 ± 0.35*

*kg* kilograms, *cm* centimeters, *m* meters, *MET* metabolic equivalents

*p ≤ 0.05

### Torque-angle curve

**Fig. 2:**
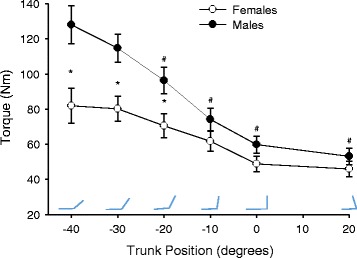
Torque-angle curve. Maximal voluntary isometric torque of trunk flexor muscles at multiple sagittal plane trunk positions for males and females. The *asterisk* denotes a significant sex difference in torque at this position (*p* < .05); the *number sign* denotes a significant difference in maximal torque (males and females collapsed) from that at the most extended position (−40°) (*p* < 0.05)

Both males and females generated larger MVC torque in extended positions (−40°, −30°, −20°) relative to more flexed positions (−10°, 0°, 20°; position: *F*_5, 27_ = 25.4, *p* < 0.001, *η*_*p*_^2^ = 0.825; Fig. 2). Pairwise comparison indicated all positions, with the exception of −30°, were statistically different (*p* < 0.05) than the position of peak torque (−40°). However, males had greater isometric torque than females (sex: *F*_1, 31_ = 7.5, *p* = 0.01, *η*_*p*_^2^ = 0.194), but not for all positions (position × sex: *F*_2, 4_ = 6.9, *p* = 0.001, *η*_*p*_^2^ = 0.182). Post hoc testing (*t* tests with adjusted *α* < 0.025) demonstrated sex differences in strength for the extended positions (−40°, *t*_31_ = −3.0, *p* = 0.006; −30°, *t*_31_ = −3.2, *p* = 0.003; −20°, *t*_31_ = −2.5, *p* = .022) with males generating greater torque than females in these positions. No sex differences in MVC strength were present in the −10°, 0°, and 20° positions.

### EMG for torque-angle curve

EMG activity of the rectus femoris did not differ across trunk position (position: *F*_5, 27_ = 1.6, *p* = 0.199, *η*_*p*_^2^ = 0.227) and was not different between sexes (sex: *F*_1, 31_ = 1.0, *p* = 0.319, *η*_*p*_^2^ = 0.032), suggesting that the differences in torque across positions was not a result of contributions from the hip flexor muscles.

### Fluctuations in torque (steadiness)

Torque steadiness was quantified for contraction intensities ranging between 5 and 70% MVC in the upright sitting position (0°). Torque produced at each target intensity increased for both males and females (intensity: *F*_4, 15_ = 60.4, *p* < 0.001, *η*_*p*_^2^ = 0.941; intensity × sex: *F*_4, 15_ = 1.2, *p* = 0.363, *η*_*p*_^2^ = 0.238), with no difference in absolute or relative torque (% MVC) between the sexes (sex: *F*_1, 18_ = 2.4, *p* = 0.142, *η*_*p*_^2^ = 0.116 and *F*_1 ,18_ = 2.0, *p* = 0.205, *η*_*p*_^2^ = 0.087, respectively).

**Fig. 3:**
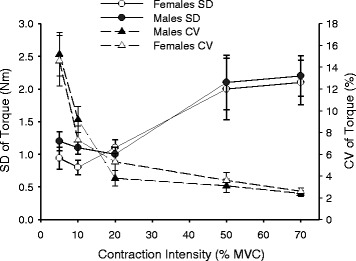
Torque steadiness of trunk flexion at different intensities of contraction. Mean (±SEM) torque fluctuations for males and females represented as the standard deviation (SD) of torque and the coefficient of variation (CV) of torque, at 5, 10, 20, 50, and 70% of maximal voluntary contraction (MVC) in upright sitting. Torque steadiness differed with contraction intensity for SD and CV, but there were no differences between males and females

Standard deviation of torque was greater at high intensities compared with low intensities (intensity: *F*_4, 15_ = 6.6, *p* = 0.003, *η*_*p*_^2^ = 0.639), for both males and females (intensity × sex: *F*_4, 15_ = 1.3, *p* = 0.299, *η*_*p*_^2^ = 0.264; Fig. 3). CV of torque was highest at a target intensity of 5% MVC and declined as target torque increased (intensity: *F*_4, 15_ = 21.4, *p* < 0.001, *η*_*p*_^2^ = 0.851) for both males and females (intensity × sex: *F*_4, 15_ = 2.5, *p* = 0.085, *η*_*p*_^2^ = 0.402; Fig. 3). There were no sex differences in SD or CV of torque (sex: *F*_1, 18_ = 0.339, *p* = 0.568, *η*_*p*_^2^ = 0.018 and *F*_1, 18_ = 0.001, *p* = 0.977, *η*_*p*_^2^ < 0.001, respectively).

### Fatigability and recovery

#### Time-to-task failure and MVC torque

Time-to-task failure for the isometric intermittent fatigue task did not differ between males and females (sex: *t*_31_ = −0.78, *p* = .440; Table 2). MVC torque was not different between males and females at baseline (57.3 ± 23.8 vs 49.5 ± 22.2, respectively; *p* = 0.336) and declined during the fatiguing exercise so that at task failure, the relative reduction in MVC torque from baseline was similar for the males and females (−30.64 ± 18.6% and −29.4 ± 13.7%, respectively; sex: *t*_31_ = 0.18, *p* = 0.862). MVC torque increased in recovery similarly for males and females (time × sex: *F*_2, 30_ = 0.6, *p* = 0.571, *η*_*p*_^2^ = 0.037) with no difference between males and females (sex: *F*_1, 31_ = 1.1, *p* = 0.313, *η*_*p*_^2^ = 0.033). By 20 min post exercise, MVC torque was fully recovered and similar between sexes (*t*_31_ = 1.0, *p* = 0.315; Table 2).

**Table 2:** Muscle function and fatigability characteristics

		Females (*n* = 18)	Males (*n* = 15)
Baseline MVC at zero position	Nm	49.5 ± 22.2	57.3 ± 23.8
Baseline MVC normalized to trunk lean mass/height from DXA	Nm/kg cm^−1^	1.6 ± 0.6	1.5 ± 0.7
Baseline MVC normalized to abdominal muscle thickness below umbilicus from US	Nm cm^−1^	51.0 ± 22.0	48.5 ± 20.3
Time-to-task failure	min	10.6 ± 5.5	12.4 ± 7.4
Rating of perceived exertion at task failure	0–10 scale	6.9 ± 2.0	6.0 ± 2.4
MVC at task failure	% baseline MVC	70.6 ± 13.7%	69.6 ± 18.6%
MVC at 10 min recovery	% baseline MVC	98.9 ± 23.7	90.5 ± 18.8
MVC at 20 min recovery	% baseline MVC	102.2 ± 18.7	94.2 ± 26.3

*MVC* maximal voluntary contraction, *US* ultrasound, *DXA* dual X-ray absorptiometry, *min* minutes

No significant differences between sexes

#### Torque and steadiness during the fatiguing task

Average torque (Nm) and relative torque (% MVC) produced during the submaximal contractions was similar between the sexes (sex: *F*_1, 31_ = 0.58, *p* = 0.454, *η*_*p*_^2^ = 0.018 and *F*_1, 31_ = 0.07, *p* = 0.797, *η*_*p*_^2^ = 0.002, respectively) and declined over time (time: *F*_4, 28_ = .7, *p* < 0.001, *η*_*p*_^2^ = 0.604 and *F*_4, 28_ = 12.4, *p* < 0.001, *η*_*p*_^2^ = 0.638, respectively) for both males and females (time × sex: *F*_4, 28_ = 0.43, *p* = 0.789, *η*_*p*_^2^ = 0.057 and *F*_4, 28_ = 0.72, *p* = 0.584, *η*_*p*_^2^ = 0.094, respectively).

**Fig. 4:**
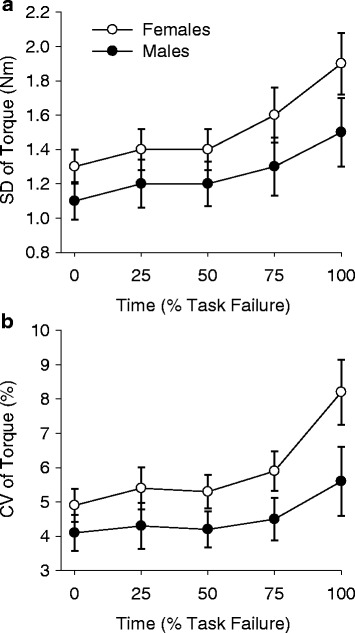
Steadiness of submaximal contractions during the fatiguing task. Steadiness of submaximal contractions performed during the intermittent isometric trunk flexion fatiguing protocol. Standard deviation (SD) of torque (**a**) and coefficient of variation (CV) of torque (**b**) are shown as the mean ± SEM of three submaximal contractions at each quartile (beginning, 25%, 50%, 75%, and end) of the total time-to-task failure. Fluctuations in torque increase over time for both males and females (*p* < 0.05) with no sex difference in steadiness between males and females

SD of torque increased during the fatiguing task (time: *F*_4,28_ = 6.1, *p* = 0.001, *η*_*p*_^2^ = 0.467) for both males and females (time × sex: *F*_4, 28_ = 0.64, *p* = 0.642, *η*_*p*_^2^ = 0.083; Fig. 4a). CV of torque also increased throughout the fatiguing protocol (time: *F*_4, 28_ = 6.4, *p* = 0.001, *η*_*p*_^2^ = 0.476) for both males and females (time × sex: *F*_4, 28_ = 0.94, *p* = 0.456, *η*_*p*_^2^ = 0.118; Fig. 4b). There was no sex difference in force fluctuations when measured with SD or CV of torque (sex: *F*_1, 31_ = 2.0, *p* = 0.168, *η*_*p*_^2^ = 0.060 and *F*_1, 31_ = 3.0, *p* = 0.094, *η*_*p*_^2^ = 0.088, respectively).

#### EMG activity during the fatiguing task

**Fig. 5:**
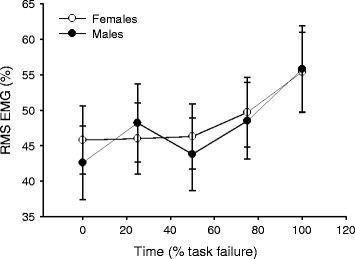
EMG of the rectus abdominis during the fatiguing task. Mean (±SEM) RMS EMG (expressed as percent of maximal RMS EMG, %) of the rectus abdominis during submaximal trunk flexion contractions across the fatiguing protocol. Rectus abdominis EMG increased over time (*p* < 0.05) for both sexes (*p* > 0.05) with no sex difference (*p* > 0.05)

EMG activity (% MVC) of the rectus abdominis (Fig. 5) and external oblique muscles increased throughout the fatiguing protocol (time: *F*_4, 28_ = 5.7, *p* = 0.002, *η*_*p*_^2^ = 0.449 and *F*_4, 28_ = 5.9, *p* = 0.001, *η*_*p*_^2^ = 0.457, respectively), similarly for males and females (time × sex: *F*_4, 28_ = 0.615, *p* = 0.432, *η*_*p*_^2^ = 0.123 and *F*_4, 28_ = 0.615, *p* = 0.655, *η*_*p*_^2^ = 0.081, respectively). There was no sex difference of rectus abdominis or external oblique EMG activity during the fatigue task (sex: *F*_1, 31_ = 0.02, *p* = 0.899, *η*_*p*_^2^ = 0.001 and *F*_1, 31_ = 2.9, *p* = 0.096, *η*_*p*_^2^ = 0.087, respectively).

Rectus femoris EMG remained low (<12% of maximal EMG) throughout the fatiguing protocol for both males and females (time × sex: *F*_4, 28_ = 1.6, *p* = 0.190, *η*_*p*_^2^ = 0.191), with no effect of time (time: *F*_4, 28_ = 1.7, *p* = 0.175, *η*_*p*_^2^ = 0.197) or sex (sex: *F*_1, 31_ = .2, *p* = 0.275, *η*_*p*_^2^ = 0.038).

### Associations between variables

**Fig. 6:**
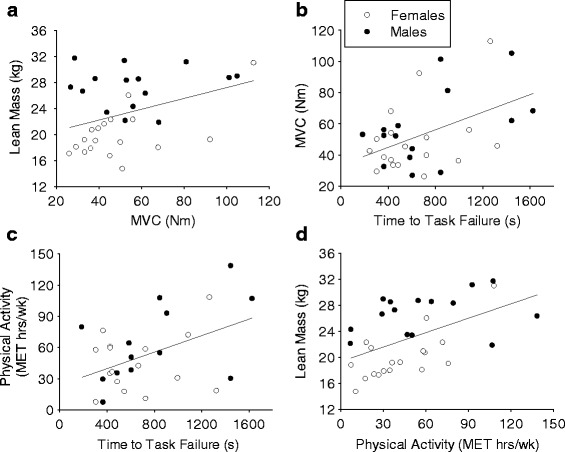
Associations. **a** Greater MVC torque of trunk flexor muscles was associated with larger lean mass in the trunk (*r* = 0.378, *p* = 0.011). **b** Longer-time-to-task failure during an intermittent isometric trunk flexion fatiguing exercise task was associated with greater MVC torque of trunk flexor muscles (*r* = 0.473, *p* = 0.005). **c** Longer-time-to-task failure of the trunk flexor muscles was associated with self-reported physical activity levels over the preceding year (*r* = 0.456, *p* = 0.030). **d** Greater self-reported physical activity levels over the preceding year were associated with greater lean mass in the trunk (*r* = 0.486, *p* = 0.007)

Trunk flexor MVC torque in upright sitting (0° trunk flexion) was positively, linearly correlated with fatigability of the trunk flexor muscles (*r* = 0.473, *r* ^2^ = 0.223, *p* = 0.005; Fig. 6a). Trunk flexor MVC torque was also positively correlated with lean mass in the trunk, and this correlation was strongest at the −40° position, where both sexes generated the greatest peak torque (−40°, *r* = 0.595, *r* ^2^ = 0.354, *p* < 0.001; 0°, *r* = 0.378, *r* ^2^ = 0.143, *p* = 0.03, Fig. 6b). Longer time to failure of the trunk flexor muscles was associated with greater self-reported physical activity over the previous 12 months (*r* = 0.456, *r* ^2^ = 0.208, *p* = 0.011; Fig. 6c). Greater self-reported physical activity over the previous 12 months was also associated with greater lean mass in the trunk (*r* = 0.486, *r* ^2^ = 0.236, *p* = 0.007; Fig. 6d).

## Discussion

There were several novel findings in this study. First, there was no sex-related difference in fatigability or steadiness of the trunk flexor muscles. Second, men were stronger than women (MVC torque), but this was only at the more extended positions. Accordingly, males had more lean mass than females (measured with DXA scan) and greater rectus abdominis muscle thickness (measured with ultrasonography). The strength of the relationship between lean mass and strength was strongest in the extended positions (−40, −30, −20), where a sex difference in strength was also observed. However, there was no sex difference in fatigability or in strength in the upright and flexed positions (−10, 0, 20). Third, MVC torque and fatigability (time-to-task failure) of the trunk flexor muscles, both performed in upright sitting, were positively correlated, such that stronger individuals were less fatigable, and this is in contrast to several other muscle groups, such as the elbow flexor muscles [28]. Physical activity levels (self-reported) were associated with fatigability, demonstrating that more physically active people were less fatigable.

The relationship between torque steadiness and contraction intensity that we observed is consistent with that seen in other muscle groups such as the plantar flexors, dorsiflexors, finger abductors, and elbow flexor muscles, such that the SD of torque increased and CV of torque decreased as contraction intensity increased [18, 20]. However, there was no sex difference in the torque steadiness during trunk flexion contractions while in the upright sitting. For both sexes, however, the CV of torque of the trunk flexor muscles (15%) was higher than that typically seen in other muscle groups at low contraction intensities (<10% MVC) (Fig. 3), such as the first dorsal interosseous (~4%), elbow flexors (~2%), and quadriceps muscles (~1.5%) [34–36]. As for other muscles, common drive to the motor units and their discharge rates of the trunk flexor muscles impact the steadiness of contraction [16], probably explaining the similarity in the shape of the CV-force intensity curve between the abdominals and limb muscles.

There are several possible explanations for these muscle group differences in torque steadiness amplitude, i.e., the greater CV of torque of the trunk flexor muscles. First, the abdominal muscles are innervated from several spinal levels (T7–L1) [2]. The activation of many alpha motor neurons from multiple spinal levels is required to control torque generated by the multiple large muscles that comprise the trunk flexors [2]. The neurological complexity of this task may contribute to the large fluctuations in torque. The large CV of torque of the trunk flexor muscles may also be impacted by the relatively long and massive trunk, which may make this body segment more difficult to control than smaller limb segments, like the forearm or index finger. Ventilation may also impact torque steadiness, as the active abdominal muscles must accommodate the rhythmic expansion and contraction of the thorax and abdomen (trunk) during strength testing. This movement of the rib cage may also reduce the stability of the proximal attachments of the abdominal muscles [37]. There may be minor contributions in the force output from chest and shoulder muscles, such as the upper trapezius muscles, because during the task, the trunk was restrained by two large straps that contact the superior aspects of the shoulders. Lastly, during contraction, the summation of forces from multiple motor units is influenced by the interaction between contractile tissue and connective tissue [38]. Thus, the presence of multiple tendinous intersections within the rectus abdominis [2], and the fascial attachments of the internal and external obliques, may impact the stability of the force generated by the muscle fibers and transferred across the connective tissue, possibly influencing the magnitude of the torque fluctuations during trunk flexion. The contribution of the mechanical and anatomical features of this unique muscle group, and the influence of discharge rate variability of the motor units from multiple muscles originating from common drive, is yet to be explored.

There was no sex-related difference in fatigability of the trunk flexor muscles for strength-matched males and females during the submaximal, intermittent isometric fatiguing protocol. This finding is in contrast to other muscles, such as the elbow flexors, where males demonstrated greater fatigability compared with strength-matched females [21]. The lack of sex difference in fatigability may be due to the fact that the abdominal muscles are a postural and ventilatory muscle group and thus may be designed to be especially fatigue resistant in both sexes. Häggmark and Thorstensson [39] showed that the abdominal muscles of males and females are comprised of approximately 55–58% type I muscle fibers, which are fatigue resistant relative to other fibers (type II). In other muscle groups, females tend to have a greater proportion of type I muscle fibers than males, which may contribute to females being more fatigue resistant than males [10, 11]. However, in muscle groups that have a high proportion of type I fibers in both males and females, such as the tibialis anterior, the sex difference in fatigability is diminished or absent [10, 40], which is consistent with our findings. Furthermore, ratings of perceived exertion at task failure were not different between sexes in our study, suggesting that males and females gave similar effort during the fatiguing exercise task (Table 2). Ratings of perceived exertion at task failure were not, on average, at maximal levels, because some participants reported feeling as though they could continue the task if allowed to utilize compensatory movement strategies. However, all participants met the criteria for failure of the fatiguing task. Importantly, our study showed that physical activity was more a determinant of fatigability than the sex of the individual, as shown by the significant correlation between time-to-task failure and self-reported physical activity.

Strength and fatigability of the trunk flexor muscles were positively correlated. This is in contrast to most other muscle groups, where weaker individuals are more fatigue resistant, such as for sustained isometric contractions of the elbow flexor and hand grip muscles in young adults [28, 34, 41], where occlusion of blood flow is the primary mechanism responsible for the inverse relationship between strength and fatigability. The current study utilized an intermittent isometric protocol for which occlusion of blood flow is not a primary mechanism, thus making it less likely that strength-related blood flow differences between participants would influence fatigability. The role of the abdominal muscles in stability of the lumbar spine and pelvis, and as accessory muscles of ventilation [2], may explain the physiological need for a positive correlation between strength and fatigability in order to minimize injury risk and to avoid possible impairments with breathing during exercise. While these mechanisms were not tested in this study, it is possible that some combination of neural input from multiple large abdominal muscles, architecture of the muscle and connective tissue [42], blood flow [43], and sympathetic drive [44] to this muscle group, may contribute to people with stronger abdominal muscles exhibiting greater fatigue resistance.

The positive correlation between strength and fatigability in this study may provide insight into the lack of a sex difference in fatigability. Females are typically weaker than males and demonstrate greater resistance to fatigue but there was no sex difference in strength for the trunk flexor muscles in upright sitting, and this was the position for the test of fatigability. In strength-matched males and females who performed an intermittent, isometric submaximal fatiguing protocol with the elbow flexors, also at 50% of MVC torque, women were less fatigable than men [21]. We did not observe this sex difference in fatigability of the trunk flexors in the males and females in this study, who did not differ in strength in upright sitting. While this study did not examine mechanisms responsible for fatigability, we hypothesize that several factors may contribute to the lack of a sex difference in fatigability. However, future research is needed to identify the mechanisms responsible for the relationship between strength and fatigability in this muscle group. This association also supports the importance of “core” strengthening.

## Conclusions

This study shows that there are no sex differences in fatigability or force control during isometric trunk flexion contractions. These findings of minimal differences in fatigability for the trunk muscles is in contrast to other studies that show clear differences in fatigability of other muscle groups, such as the elbow flexors and knee extensors [11]. Furthermore, although men were stronger than females in the extended trunk positions of sitting, there was a minimal difference in maximal strength in upright and flexed sitting positions. Stronger males and females during upright sitting, however, were less fatigable than weaker individuals, and both strength and fatigability may be modulated by physical activity levels. The relationship between strength and fatigability of the trunk flexor muscles and physical activity supports the importance of abdominal muscle strengthening to offset fatigability.

## Abbreviations

ANOVA: Analysis of variance
BMI: Body mass index
CV: Coefficient of variation
DXA: Dual X-ray absorptiometry
EMG: Electromyography
IAP: Intra-abdominal pressure
MET: Metabolic equivalents
MVC: Maximal voluntary contraction
Nm: Newton meters
RMS: Root mean square
SD: Standard deviation
SEM: Standard error of the mean
